# Nuclear and Chloroplast Microsatellites Show Multiple Introductions in the Worldwide Invasion History of Common Ragweed, *Ambrosia artemisiifolia*

**DOI:** 10.1371/journal.pone.0017658

**Published:** 2011-03-10

**Authors:** Myriam Gaudeul, Tatiana Giraud, Levente Kiss, Jacqui A. Shykoff

**Affiliations:** 1 UMR CNRS 7205 ‘Origine, Structure et Evolution de la Biodiversité’, Muséum National d'Histoire Naturelle, Paris, France; 2 UMR CNRS 8079 ‘Ecologie, Systématique et Evolution’, Université Paris-Sud, Orsay, France; 3 Department of Plant Pathology, Plant Protection Institute of the Hungarian Academy of Sciences, Budapest, Hungary; University Copenhagen, Denmark

## Abstract

**Background:**

*Ambrosia artemisiifolia* is a North American native that has become one of the most problematic invasive plants in Europe and Asia. We studied its worldwide population genetic structure, using both nuclear and chloroplast microsatellite markers and an unprecedented large population sampling. Our goals were (i) to identify the sources of the invasive populations; (ii) to assess whether all invasive populations were founded by multiple introductions, as previously found in France; (iii) to examine how the introductions have affected the amount and structure of genetic variation in Europe; (iv) to document how the colonization of Europe proceeded; (v) to check whether populations exhibit significant heterozygote deficiencies, as previously observed.

**Principal Findings:**

We found evidence for multiple introductions of *A. artemisiifolia*, within regions but also within populations in most parts of its invasive range, leading to high levels of diversity. In Europe, introductions probably stem from two different regions of the native area: populations established in Central Europe appear to have originated from eastern North America, and Eastern European populations from more western North America. This may result from differential commercial exchanges between these geographic regions. Our results indicate that the expansion in Europe mostly occurred through long-distance dispersal, explaining the absence of isolation by distance and the weak influence of geography on the genetic structure in this area in contrast to the native range. Last, we detected significant heterozygote deficiencies in most populations. This may be explained by partial selfing, biparental inbreeding and/or a Wahlund effect and further investigation is warranted.

**Conclusions:**

This insight into the sources and pathways of common ragweed expansion may help to better understand its invasion success and provides baseline data for future studies on the evolutionary processes involved during range expansion in novel environments.

## Introduction

Invasive species offer useful models for studying rapid range expansion in novel environments, which can imply pre-adaptation, phenotypic plasticity or adaptation. Evolutionary aspects of biological invasions have long been neglected, with most past focus being on ecological aspects, but recently, interest in the evolution of invasive species has grown (e.g. [1]–[4]). However, knowledge of population history and historical relationships is a prerequisite for examining the evolution of phenotypic traits that may be subject to selection in the new environment. In particular, one needs to identify the most likely source populations/regions for the invasion and determine whether there were single or multiple introduction events. Have invasive populations undergone a genetic bottleneck? What are the pathways of introduction? What is the extent of contemporary gene flow? Such information is also crucial for understanding the success of invasive species, documenting their colonization modes, and designing measures to limit their expansion (e.g. biological control).

Species introductions are sampling events and should therefore generate genetic bottlenecks. In agreement with this prediction, loss of variation is a frequent, although not ubiquitous, feature of introductions [5]. Multiple introductions can occur, either at the population level (i.e. one population resulting from introductions from several native populations) or at the regional level (i.e. each invasive population being founded from a different single source population). Such multiple introductions can lead to levels of genetic diversity as high in introduced as in native populations (or regions, respectively), and has been shown in numerous case studies (e.g. [6]–[8]). In the introduced range, founder effects tend to increase among-population differentiation and separate introductions may establish differentiated gene pools in different sites through founder effects, subsequent drift and/or responses to selection [9], [10]. On the contrary, repeated introductions into some sites from different sources may convert among-population variation in the native range into within-population variation in the introduced one, thereby decreasing among-population differentiation (e.g. [11]–[13]). Recent range expansion and gene flow can also homogenize allele frequencies. Indeed empirical studies of invasive plants reveal that genetic differentiation among invading populations is often diminished relative to differentiation in the native range [14].

One of the most problematic invasive plants in Europe and Asia is *Ambrosia artemisiifolia* L. (Asteraceae; also called common ragweed). This wind-pollinated monoecious annual is a common native of North America, and has been introduced to South America, Europe, Asia and Australia, where it has become invasive [15], [16]. It is a successful pioneer and grows abundantly in disturbed habitats, including cultivated fields, roadsides and railways, river banks, construction sites and waste places, on a variety of soil types. *A. artemisiifolia* causes large economic losses by reducing crop yields in agricultural fields (e.g. soybean, sunflower), represents a significant challenge to the management of natural resources [17], and its massive production of pollen often causes serious allergic problems for humans [18]. Although *A. artemisiifolia* is a self-incompatible annual species ([19]; but see Discussion) with no vegetative propagation [15], three main characteristics may explain its success as an invader [20]: enormous production of wind-borne pollen assuring pollination success even of isolated individuals, high fecundity (large plants can produce up to 62000 seeds [15]) and long-term seed dormancy (at least 20 years [21]).

Highly infested countries include France (especially the Rhone valley), Italy (especially the Po valley), Hungary and Russia (North Caucasus, Krasnodar territory [22]). In Hungary, about 80% of arable land is colonised and 20% of the population suffers pollen allergies [18], [23]. Ragweed is also found in South America, China (mostly in the Eastern part of the country [24]), Australia (mostly along the Eastern coast, across New South Wales and Queensland [25], [26]), Japan and Korea [27]. In almost all cases, the species was detected as early as 1900–1950 (or even before), but its explosive spread occurred after 1950 (e.g. [22], [24]–[26], [28]).

Both herbarium records [28] and recent molecular studies [29], [30] based on nuclear microsatellite markers suggested multiple independent introductions from North America into France. The main mechanism of ragweed dispersal is probably contamination of crop seed lots (e.g. cereals, sunflower [28]). Global trade (together with inter-continental travel) has indeed been shown to have a major role in the increasing numbers of biological invasions over the last decades [31], [32]. Other origins are also suspected, such as contaminated bird food (in urban areas) and forage, ship ballast, and military movements [16], [20], [28]. Once the species is established, the achenes of *A. artemisiifolia* are mostly dispersed by human activities (achenes do not possess any obvious morphological dispersal mechanism). In several instances, the massive spread of common ragweed has been correlated with major socio-economic transitions that increased the area of disturbed or fallow land, such as during the communist economy (1948–1989) in Eastern Europe, when many sites e.g. extensive border areas and military zones were left uncultivated [23], [33] and the political transitions to young democracies in Eastern Europe, with the closure and cessation of cultivation of many agricultural co-operatives [23], [34]. The extensive waste lands generated by the war in former Yugoslavia [16] also probably favoured ragweed expansion. Finally, European common agriculture policies may contribute to some extent to colonization by ragweed when arable land in low-productivity areas is abandoned, creating new suitable habitats for weed expansion [16], [34].

To gain insights into the historical relationships among *A. artemisiifolia* populations from the native and introduced ranges, and to shed light on the colonization history of this worldwide invader, we investigated the neutral genetic structure of this species, expanding previous samplings [30], [35] to include additional invaded regions and native populations. In the invasive range, Genton et al. [30] previously surveyed French populations and Gladieux et al. [35] later studied six additional populations from Eastern Europe. We sampled Europe as continuously as possible, and also studied several populations from South America, Asia and Australia. Moreover, in the native range, earlier studies [30], [35] focused on eastern North America whereas we included populations from western North America, therefore covering a larger geographic area and adding potential, hitherto unexplored, source populations.

In France, Genton et al. [30] found high within-population diversity, low among-population differentiation and no pattern of isolation by distance, indicating that introduced populations probably resulted from a mixture of different native populations. They also observed a cline in diversity away from the putative initial area of introduction, suggesting that range expansion occurred through sequential bottlenecks from the original populations, and not from subsequent new introductions. Gladieux et al. [35] suggested that Eastern European populations did not originate from the earlier established French populations but rather represented multiple independent introductions from other sources, or introductions from an unidentified highly diverse native population. At the population level, previous studies reported high levels of heterozygote deficiency relative to Hardy-Weinberg equilibrium and null alleles were invoked to explain this result [29], [30], [35].

We addressed the following questions: (i) Can we identify the sources of the different invasive populations in the world, especially of the previously unanalyzed populations in Australia, China and South America? Can we confirm that Eastern European populations originated from other sources than Western European populations? And if so, from where did they originate? (ii) Were all invasive populations founded by multiple introductions? (iii) How have the introductions affected the amount and structure of genetic variation in Europe (compared to the patterns observed in the native range)? (iv) In Europe, did the expansion proceed in a stepwise manner, each population being colonized by a neighboring population, or as a result of long-distance dispersal within the continent, or did populations result from independent colonization events from the native range? (v) Do populations exhibit significant heterozygote deficiencies? And if so, can we suggest plausible explanatory mechanism(s)?

We used both nuclear and chloroplast microsatellite markers, that differ in their mode of inheritance (biparental vs. maternal only) and mutation rate (higher at nuclear markers [36]) and therefore give complementary insights into the invasion history and population dynamics of the species.

## Materials and Methods

### Plant material

**Figure 1:**
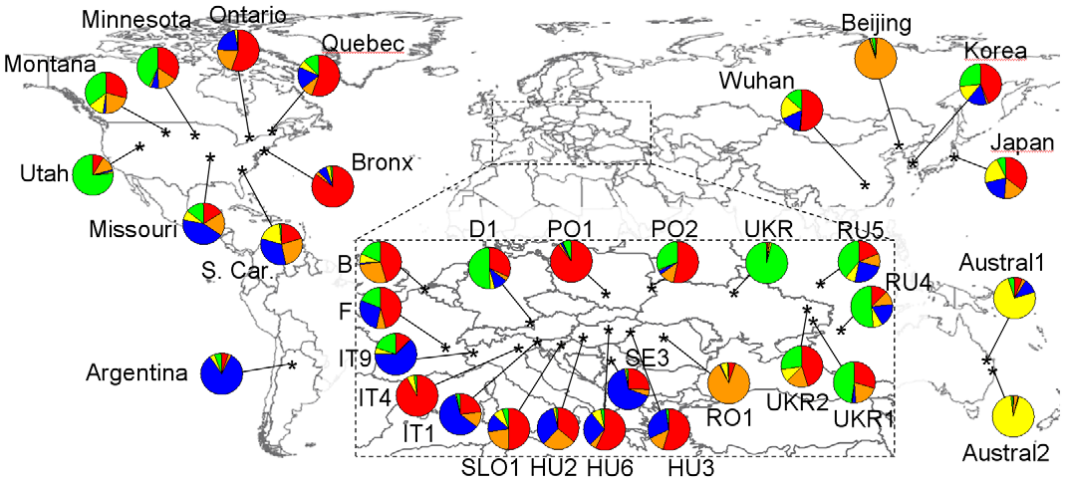
Map and nDNA genetic composition (based on Structure) of the studied populations. For each population, the pie represents the membership coefficients to the five clusters inferred in Structure.

Leaf material was collected from 32 natural populations: eight from North America, 19 from Europe (including Ukraine and Russia), one from Argentina, two from China and two from Australia (Table 1, Fig. 1) during summer 2007 and 2008. Twenty plants, located 2–5 m apart from each other, were sampled from each site (except Bronx, Argentina and UKR where only 11, 16 and 18 samples were available, respectively). Most populations covered areas of at least one thousand square meters, and counted several hundred to several thousand plants. Leaf samples were dried in silica gel and stored at room temperature until DNA extraction. Moreover, material from herbarium specimens collected in Japan (seven samples; Takamatsu n°145, 398, 523, 1266, 2733, 3656, 3897) and Korea (two samples; Shin n°19892, 19613) and respectively stored at the Mie University Mycological Herbarium (Tsu, Japan) and Mycological Herbarium of the Korea University (Seoul, South Korea) was included in the analyses, leading to a total of 634 individuals.

**Table 1:** Geographic and genetic characteristics of the studied populations.

Population name	Country	Latitude	Longitude	Nb samples (nDNA)	AR	*H*_e_	*F*_IS_	Nb samples (cpDNA)	Nb hapl	Nb private hapl	Mean nb of pairw diff among indiv
Utah	USA	41°44′44″ N	111°54′41″ W	20	6.00	0.687	0.291	20	3	1	1.326
Montana	USA	46°59′24″ N	104°10′48″ W	20	6.27	0.771	0.373	20	3	0	3.200
Minnesota	USA	45°52′52″ N	95°22′31″ W	20	7.23	0.813	0.351	20	6	0	1.837
Missouri	USA	38°41′05″ N	90°18′45″ W	20	7.40	0.802	0.256	18	4	0	1.843
S. Car.	USA	33°59′18″ N	81°01′34″ W	20	6.35	0.760	0.350	19	4	1	0.807
Ontario	Canada	44°23′04″ N	78°23′00″ W	20	7.13	0.814	0.261	20	8	2	2.732
Quebec	Canada	46°49′27″ N	71°13′38″ W	20	7.47	0.792	0.295	19	2	0	0.199
Bronx	USA	40°51′28″ N	73°54′36″ W	11	5.71	0.724	*0,193*	11	1	0	0.000
B	Belgium	50°55′25″ N	03°12′48″ E	20	5.24	0.740	0.350	19	6	0	3.813
F	France	45°43′57″ N	04°59′49″ E	20	6.97	0.798	0.262	18	6	1	2.320
D1	Germany	47°49′14″ N	12°49′36″ E	20	5.59	0.725	0.188	19	5	1	1.532
IT9	Italy	45°21′36″ N	07°50′06″ E	20	5.77	0.742	0.356	19	4	0	0.901
IT4	Italy	45°28′30″ N	11°43′48″ E	20	5.08	0.719	*0,090*	20	1	0	0.000
IT1	Italy	45°54′11″ N	13°31′16″ E	20	6.42	0.784	0.245	20	3	1	3.705
SLO1	Slovenia	45°54′36″ N	15°27′00″ E	20	7.42	0.829	0.331	20	7	0	2.811
HU2	Hungary	46°37′48″ N	17°17′24″ E	20	7.04	0.794	0.400	18	5	1	2.373
HU6	Hungary	47°20′51″ N	19°26′56″ E	20	7.37	0.825	0.179	20	6	0	3.226
HU3	Hungary	47°07′27″ N	21°45′33″ E	20	6.85	0.773	0.225	19	2	0	0.561
SE3	Serbia	44°58′44″ N	19°36′57″ E	20	6.29	0.772	0.343	18	4	0	2.477
PO1	Poland	50°01′40″ N	20°00′33″ E	20	5.62	0.694	0.327	19	4	0	2.550
PO2	Poland	51°10′00″ N	23°47′60″ E	20	6.16	0.782	0.446	17	5	0	2.956
RO1	Romania	46°32′34″ N	24°34′02″ E	20	4.92	0.709	0.244	19	4	0	1.509
UKR	Ukraine	50°43′00″ N	30°51′00″ E	18	4.40	0.634	0.400	18	1	0	0.000
UKR1	Ukraine	48°05′55″ N	37°49′52″ E	20	6.66	0.766	0.190	20	5	0	3.026
UKR2	Ukraine	49°01′38″ N	37°32′10″ E	20	6.64	0.798	0.359	19	4	0	2.807
RU4	Russia	46°20′17″ N	42°07′50″ E	20	5.89	0.760	0.360	20	3	0	1.037
RU5	Russia	50°58′26″ N	39°30′46″ E	20	6.83	0.769	0.271	20	4	0	2.905
Argentina	Argentina	26°48′43″ N	65°18′04″ W	16	5.62	0.784	0.411	16	3	2	3.950
Wuhan	China	29°09′54″ N	113°12′32″ E	20	5.94	0.727	0.331	19	3	1	1.930
Beijing	China	41°36′10″ N	123°48′41″ E	20	4.80	0.734	0.422	20	2	0	1.437
Japan	Japan	34°59′43″ N	135°51′23 E	7	-	-	-	5	3	1	-
Korea	Korea	37°33′59″ N	126°58′40'' E	2	-	-	-	2	2	1	-
Austral1	Australia	27°14′26″ S	152°25′19″ E	20	5.43	0.771	0.522	19	3	1	0.561
Austral2	Australia	28°23′37″ S	153°24′13″ E	20	4.65	0.670	0.274	20	1	0	0.000
Mean_North America (± S. D. among populations)					6.70±0.69	0.770±0.045	0.296±0.060		3.87±2.23		1.49±1.14
Mean_Europe (± S. D. among populations)					6.17±0.87	0.759±0.048	0.293±0.093		4.16±1.64		2.13±1.18
Mean_non European invasive populations (± S. D. among populations)					5.29±0.55	0.737±0.045	0.392±0.095		2.40±0.89		1.58±1.52

Populations are grouped into three spatial groups (North America, Europe and non-European invasive populations) and roughly ordered from West to East. Nb samples, number of samples; AR, allelic richness (based on the minimal sample size of 8 individuals); *H*_e_, expected heterozygosity; *F*_IS_ estimates in italics were not significant; Nb hapl, number of haplotypes; Mean nb of pairw diff among indiv, mean number of pairwise differences among individuals.

### Microsatellite procedure

DNA was extracted using the DNeasy 96 Plant Kit (QIAGEN). We used a total of nine nuclear and four chloroplast microsatellite markers: three nuclear microsatellite markers (Amb12, Amb30 and Amb82) developed by Genton et al. [30], six nuclear markers (Ambart04, Ambart06, Ambart09, Ambart13, Ambart21, Ambart27) described by the Molecular Ecology Resources Primer Development Consortium [37], one universal chloroplast locus (NTCP9 [38]) and three chloroplast markers located in the trnC-ycf6 and rps16 regions, for which we developed primers (c6T448_F: GAT TGG ATA GCC GGC AGA TA; c6T448_R: TTC CTT TTT CTT GGC CTT CA; s16T148_F: AGC CGT TCC AAC AAA TGA AA; s16T148_R: AAA CGA TGT GGT ARA AAG CAA C; s16T690_F: ACT CAT AGT CCT TTT TAT TTA GCT TCC; s16T690_R: TTT GAG AAT TAT TGA ACT TGA GTT ATG). We checked by direct sequencing that all differences between cpSSR size variants were due to variable numbers of mononucleotide repeats.

Multiplex PCRs were performed, amplifying several loci simultaneously. The 16 µl reaction mix contained 1 µl DNA template, 1X Taq Buffer, 2 mM MgCl2, 0.2 mM of each dNTP, varying concentrations of primers (see below; one primer per pair was fluorescently labelled), and 0.4 U Taq polymerase per primer pair included in the reaction. Primer concentrations were experimentally determined so that the intensity of all microsatellites was high enough to prevent allelic drop-out and allow unambiguous genotyping. For multiplex1, primer concentrations were 0.30 µM for Amb82, Ambart04 and Ambart13, and 0.08 µM for c6T448 and s16T690. For multiplex2A, primer concentrations were 0.60, 0.16 and 0.06 µM for Amb12, Ambart27 and s16T148, respectively. And for multiplex2B, primer concentrations were 0.10, 0.30, 0.30 and 0.20 µM for Ambart06, Ambart09, Ambart21 and NTCP9, respectively. The reaction profile was the following: 40 cycles of denaturation at 95°C for 30 s, hybridization at 50°C (for multiplex1) or 52°C (for multiplex2A and multiplex2B, respectively) for 30 s, and elongation at 65°C for 4 min, followed by a final elongation step of 10 min at 72°C. Locus Amb30 was amplified separately using 2.5 mM MgCl2, 0.2 mM of each dNTP, 0.2 µM of each primer and 0.5 U Taq polymerase and the following reaction profile: 40 cycles of denaturation at 95°C for 30 s, hybridization at 50°C for 30 s, and elongation at 72°C for 30 s, terminated by an elongation step of 10 min at 72°C. The PCR product was then mixed with multiplex 2A in a 1∶1 ratio. Finally, the internal size standard LIZ500 was added to all samples prior to loading on an automated sequencer. This final step was performed by a private genotyping company (Genoscreen, Lille, France).

Microsatellite profiles were manually genotyped using GeneScan 3.7 and Genotyper 3.7. Reproducibility was checked by performing the amplification and genotyping steps on 30 samples twice, leading to 30×9 = 270 sample × locus duplicates.

### Statistical analyses

For both nuclear and chloroplast loci, samples from Japan and Korea were discarded from population-level computations because they were not grouped into discrete natural populations and not in sufficient number to allow reliable statistical inferences.

#### Nuclear microsatellites

Within each population, linkage disequilibrium was tested between loci based on random permutations of genotypes performed with the software FSTAT [39] and followed by a Bonferroni correction for multiple tests. Genetic diversity was estimated as allelic richness (mean number of alleles per locus based on the minimal sample size [40]) and expected heterozygosity using FSTAT. Genetic structure was quantified by within-population *F*_IS_ and among-population *F*_ST_ indices using FSTAT. The statistical significance of *F*_IS_ was assessed by 5760 random permutations of alleles in each population at each locus, followed by a Bonferroni correction for multiple tests. To detect signs of recent bottlenecks, we examined deviations in heterozygosity from mutation–drift equilibrium in each population with the software Bottleneck [41]. The loss of rare alleles in recently bottlenecked populations leads to an excess of heterozygosity relative to the expected heterozygosity with the same number of alleles at mutation–drift equilibrium [41]. We assumed that microsatellite loci follow a two-phase mutation model (intermediate between the IAM and SMM models) with 70% single-step mutations and 30% multiple-step mutations. We used the implemented Wilcoxon test, which is considered the most powerful and robust among the tests proposed in Bottleneck, and we corrected the results by a Bonferroni procedure. Among-population differentiation was quantified with *F*_ST_ indices both at the global scale and among all pairs of populations. We computed the 95% confidence interval of the global *F*_ST_ by bootstrapping over loci. The overall differentiation of each population was estimated as the mean pairwise *F*_ST_ between each population and all others. Exact tests of population differentiation were also performed among all pairs of populations using Genepop [42], [43]. Pairwise differences in expected heterozygosity, allelic richness, *F*_IS_ and *F*_ST_ among North America, Europe (including Ukraine and Russia) and non-European invasive populations (Argentina, Beijing, Wuhan, Austral1 and Austral2) were assessed using permutation tests in FSTAT (for *F*_ST_ indices, we only compared North America and Europe because they cover similar geographic areas; non-European invasive populations were much more distant from each other, which would induce a bias).

To identify the potential sources of invasive populations, we attempted to assign all sampled individuals from invasive populations to their most probable source population among the sampled North American populations. We adopted the method of Rannala & Mountain [44], which uses Bayesian criteria for likelihood estimation. The probabilities of assignment were calculated following Paetkau et al. [45] based on 10,000 simulated individuals. These calculations were performed using the GeneClass 2.0.h software [46].

Based on the matrix of pairwise *F*_ST_ indices, the genetic similarity of populations was summarized using a Principal Coordinate Analysis, performed in NTSYS ([47]; the analysis included double-centring the matrix and computing eigen-vectors using the Dcenter and Eigen modules, respectively). A hierarchical analysis of molecular variance (AMOVA) was conducted to partition the total genetic variance in among-region, among-population within region, and among-individual within population components using Arlequin [48]. For this analysis, we considered two regions: North America and Europe. We tested the pattern of isolation by distance within these two regions by performing Mantel tests with 10000 random permutations to compare the genetic and geographic distance matrices.

We used several Bayesian algorithms implemented in Structure [49], [50], Instruct [51] and Structurama [52], to cluster individuals into genetically distinct groups.

- Structure uses Markov chain Monte Carlo (MCMC) algorithms to group individuals in clusters (where the numbers of clusters must be set a priori) that deviate neither from Hardy–Weinberg nor linkage equilibrium within each cluster. It also calculates the posterior probability of the data given the inferred clustering. Structure was run 20 times for each K-value from one to seven to check the consistency of the results across runs. Each run comprised a burn-in period of 200000 iterations followed by 10^6^ iterations. We adopted the admixture model, the correlated allele frequencies model, and we used sampling locations as prior information to assist the clustering (LOCPRIOR option). Hubisz et al. [53] showed that this option improves the performance of the clustering when the signal of structure is weak, but does not tend to find structure when none is present. We plotted the relationship between the K-value and (i) the probability of the data lnP(D) and (ii) as recommended by Evanno et al. [54], the ad hoc statistic ΔK which corresponds to the change of lnP(D) between consecutive K-values. We identified the most relevant number of clusters (K) as the one that maximized lnP(D) and/or ΔK, following Evanno et al. [54]. For each K-value, the similarity among runs (in terms of individual assignment to the K clusters) was estimated with Structure-sum-2009 [55] and the most likely inferred clustering was graphically displayed with Distruct [56]. Structure was also run in a similar fashion within North America and within Europe, to compare how the genetic diversity was geographically structured in the two ranges.
- Unlike Structure, which requires running the program several times under different K-values and then determining the best value *post-hoc*, Structurama employs a prior distribution of K to determine the most appropriate K-value. At each run, it also outputs posterior probabilities of each possible K-value and the mean partition, i.e. a partitioning of individuals among clusters that minimizes the squared distance to the sampled partitions across generations of the MCMC [52]. The program was run three times for each of three prior models (i.e. nine runs in total). The number of clusters and the alpha parameter were considered random variables, with the alpha parameter following a gamma probability distribution. The shape and scale (a, b) of this distribution were consecutively set to (1, 2), (2, 2) and (3, 2), respectively, corresponding to prior K-values of 4.1±2.8, 6.9±5.2 and 9.4±7.3, respectively. Each run comprised 20000 generations that were discarded as burn-in and 180000 generations that were sampled every 50 generations.
- The Bayesian approach of Instruct is very similar to that of Structure, but Instruct allows inbreeding and estimates inbreeding coefficients (that are similar to within-population *F*_IS_ indices) within the inferred clusters [51]. The approach of Instruct may be biologically more suited to *A. artemisiifolia* since we detected significant departures from Hardy-Weinberg equilibrium in almost all populations (see Results). We conducted five runs per K-value spanning from one to 10, with each run comprising 100000 iterations burn-in followed by 500000 iterations that were sampled every 50 generations (thinning).

#### Chloroplast microsatellites

Because there is no recombination within the cpDNA molecule, alleles found at all cpSSR loci were combined to compose a unique chloroplast haplotype for each individual. Individuals with missing data (n = 24) were discarded from the inference of multilocus haplotypes and from the statistics based onto these haplotypes.

First, we considered all multilocus haplotypes to draw a median-joining network based on the number of mutations among all pairs of haplotypes using the software NETWORK [57]. We adopted a two-step procedure to reduce the potential impact of homoplasy: based on an initial network, the loci were inversely weighted by the number of mutations occurring at each of them, in a second run, as recommended by Bandelt et al. [57], [58].

Within populations, we computed the number of haplotypes, number of private haplotypes (found in only one population) and mean number of pairwise differences among individuals using the software Arlequin. For this, we coded cpSSR data in a binary way, representing for each locus the number of repeats of the largest variant with ‘1’s and replacing the absent repeats of shorter variants with ‘0’s. Permutation tests (in FSTAT) and non parametric Mann-Whitney tests were performed to detect any significant difference in number of haplotypes and mean number of pairwise differences among individuals, respectively, between North America, Europe and non-European invasive populations The program SpaGeDi [59] was used to compute global and pairwise *F*_ST_ and *N*_ST_ indices of among-population differentiation based on unordered and ordered haplotypes, respectively. The input dataset contained, for each individual, the multilocus haplotype displayed. For the estimation of *N*_ST_ *'*s, the distance between haplotypes was calculated as the sum of their absolute length differences across the four loci. We performed 10000 permutations of rows and columns of the distance matrix between haplotypes to test whether *N*_ST_ > *F*_ST_. Such a significant relationship suggests that distinct haplotypes are more related within populations than among them, i.e. that genetic structure displays a significant geographic trend [60]. We conducted a Principal Coordinate Analysis (using NTSYS) based on *N*_ST_ indices, and an AMOVA of haplotype frequencies, implemented in Arlequin, to assess the proportion of genetic variance found at the region (North America vs. Europe), population and individual levels. We also performed Mantel tests (using FSTAT) within North America and Europe, based on *N*_ST_ indices_._

## Results

Reproducibility was high, with 97.7% of all sample × locus duplicates carrying the same genotype. We did not find any evidence of linkage disequilibrium between pairs of nuclear microsatellite markers.

### Genetic diversity

We detected a mean (± S. D. among loci) of 19.7 ± 8.2 alleles per nuclear microsatellite locus (spanning from 6 to 29 alleles per locus; Table 1) and a mean (± S. D. among loci) of 4.8±1.5 alleles per chloroplast microsatellite locus (spanning from 3 to 7 alleles per locus; Table 1).

At nuclear loci, expected heterozygosity was quite similar across populations whereas mean allelic richness was more variable (Table 1). At the regional level, mean allelic richness (± S. D. among loci) was 15.19±6.19, 14.92±6.42 and 13.35±5.85 alleles per locus in North America, Europe and non-European invasive populations, respectively, based on a minimum sample size of 53 individuals. All invasive populations (except B, PO1 and Wuhan) displayed at least one allele that was absent from North American populations. These alleles were most often found in several populations, but usually at very low frequency (<0.1). Only IT9, UKR, and Austral2 exhibited alleles (one allele each) that were absent from America and present at frequencies higher than 0.2 within populations. Only two populations, PO2 and Beijing, showed significant excess of heterozygosity (after the Bonferroni correction, P = 0.031 for both populations), which suggests a recent bottleneck.

CpDNA microsatellites allowed the definition of 33 multilocus chloroplast haplotypes. (Table S1). Fourteen haplotypes were private to one population, but only four of them were found in at least two individuals: haplotype K was observed in the population from Utah (in 11 individuals), haplotype D in IT1 (in 11 individuals) and haplotypes L and W were only observed in the population from Argentina (in 9 and 5 individuals, respectively; Table S1). In total, we observed 19 haplotypes in North America, 23 haplotypes in Europe (15 of which were shared with North America) and 14 haplotypes in the non-European invasive populations (8 of which were shared with North America and Europe; Table S1). Using the rarefaction method of El Mousadik & Petit [40] to account for different sample sizes across regions, estimates of haplotype richness were 18.6, 20.3 and 14.0 haplotypes for North America, Europe and non-European invasive populations, respectively, based on a minimum sample size of 101 individuals.

Permutation tests showed that North American and European populations did not differ statistically in terms of diversity (allelic richness, expected heterozygosity, number of haplotypes per population, mean number of pairwise differences among individuals; Table 1; all P>0.1). In contrast, the group of non-European invasive populations (Argentina, Wuhan, Beijing, Austral1, Austral2) was less diverse than North America and Europe in terms of allelic richness (Table 1; P = 0.004 and 0.044, respectively). The group of non-European invasive populations was not significantly different from North America but marginally less diverse than Europe for the number of haplotypes (Table 1; P = 0.147 and P = 0.051, respectively). North American, European and non-European invasive populations were not significantly different in terms of expected heterozygosity and mean number of pairwise differences (Table 1; all P>0.1).

**Figure 2:**
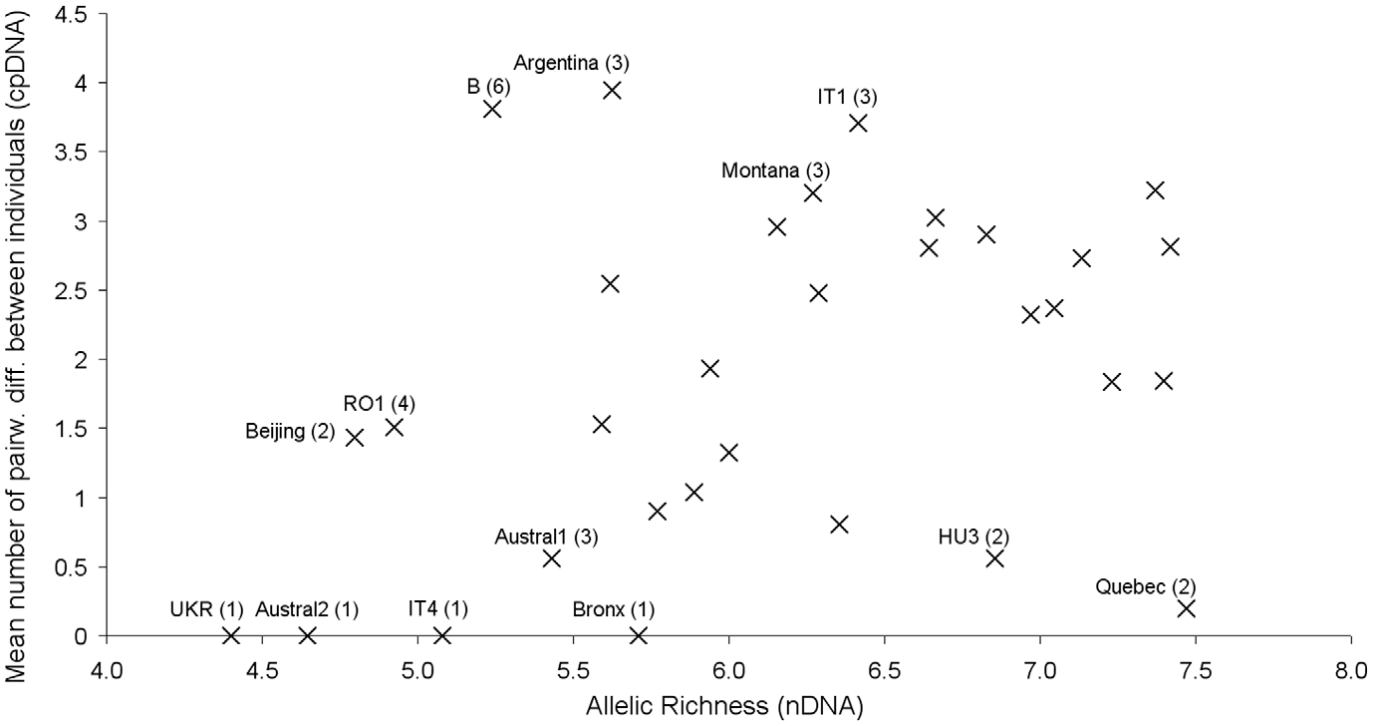
Figure 2. Within-population genetic diversity. Relationship between nDNA allelic richness and cpDNA mean pairwise number of differences between individuals (the number of haplotypes in the population is indicated within brackets). The correlation was significant (P = 0.041).

Most populations that were characterized by low estimates of nuclear allelic richness also displayed few chloroplast haplotypes and/or low mean number of pairwise differences among individuals (Fig. 2; the correlation was significant, P = 0. 041) e.g. UKR, Austral2, IT4, and Bronx.

### Genetic Structure - Bayesian Clustering (nuclear Microsatellites Only)

**Figure 3:**
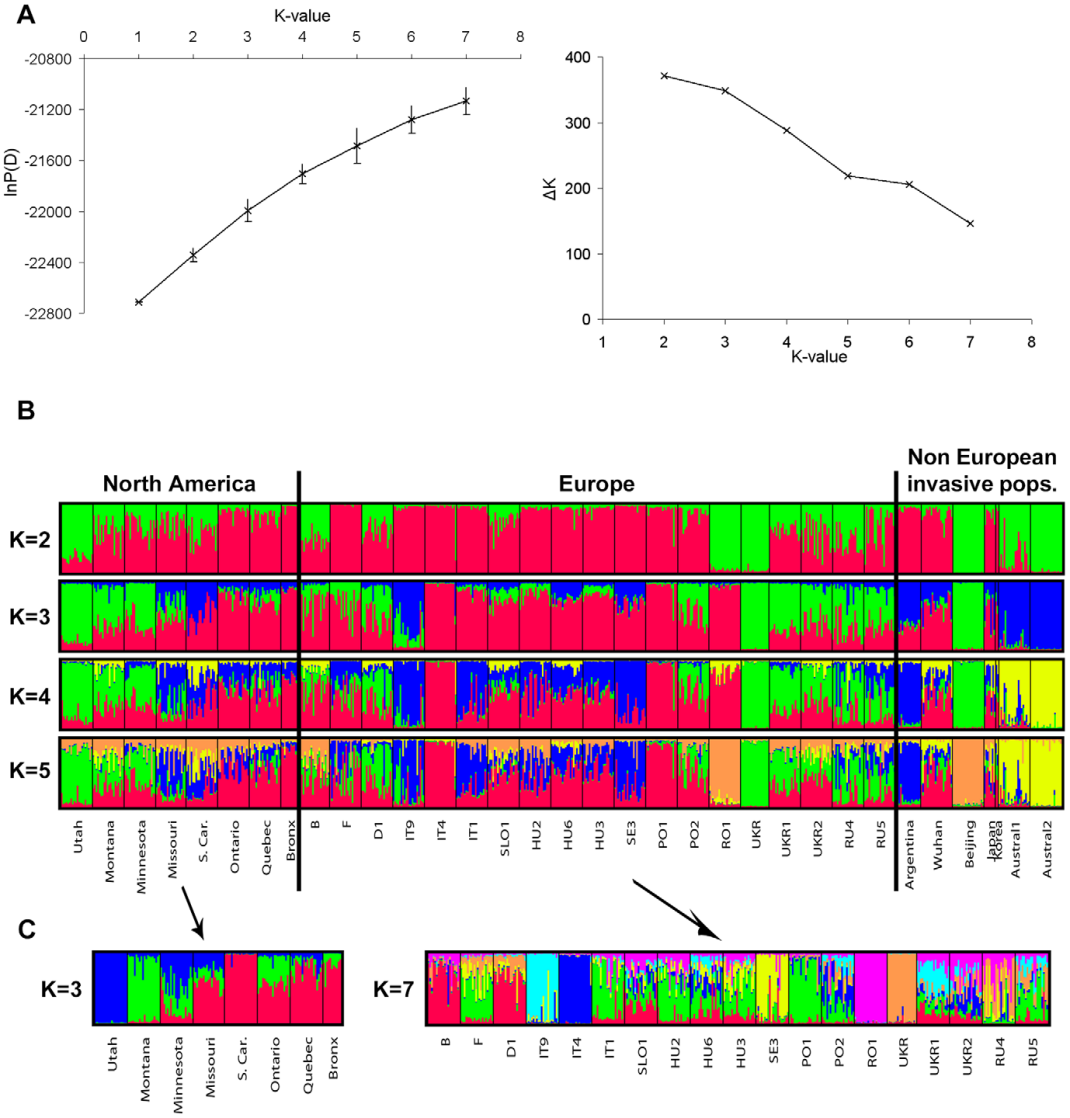
Figure 3. Bayesian analysis performed in Structure. A) on the overall nDNA dataset, relationship between K, lnP(D) and ΔK. B) On the overall nDNA data set, cluster partitioning of the populations at consecutive K-values from K = 2 to K = 5. Each vertical line represents one individual and the colors represent the membership coefficients to the K clusters. The clustering solutions inferred by Instruct and Structurama were highly similar. Colors are the same as in Fig. 1. C) On the North American (K = 3) and European (K = 7) datasets.

Out of the nine runs of Structurama, seven indicated K = 6 as the most relevant number of clusters (i.e. within each run, K = 6 was associated to the highest probability, averaging P = 0.44±0.02 across runs) and two indicated K = 7 (with P = 0.40±0.01). However, six of the nine mean partitions that were inferred counted five clusters. In the three remaining runs, the sixth cluster was a subdivision of a pre-existing cluster and was present in very low proportions in several populations. Using Structure and Instruct, the probability of the data lnP(D) steadily increased, and the change of probability between consecutive K-values (ΔK) steadily decreased when assuming increasing K values (Fig. 3A). However, we observed that from K = 6 upwards, additional clusters did not individualize additional populations but were rather represented in moderate proportions in many populations, therefore probably not revealing a genuine population genetic structure. Therefore, we did not run Structure assuming higher K-values.

At each K-value, most runs of Structure were consistent in terms of individual assignment to the K clusters (similarity ≥ 0.75). At K = 2, an East-West cline of cluster assignment was observed in North America, and the predominant cluster in western North America (in green; Fig. 3B) was also found in Eastern Europe, Beijing and the two Australian populations. At K = 3, the third cluster (in blue) was mostly represented in Australia, Italy (IT9) and Argentina while the second cluster (in green) was mostly observed in western North America and Eastern Europe. At K = 4, the Australian populations (in yellow) were again separated and, at K = 5, populations from Romania and Beijing were grouped together into a new cluster (orange). As before, we also observed some genetic similarity between western North America and Eastern Europe (Ukraine and Russia) on the one hand, and eastern North America and Western and Central Europe (IT9 to PO1) on the other hand. Instruct allowed estimation of inbreeding levels spanning from 0.28 to 0.40 within each of the five inferred clusters.

Within North America, the highest lnP(D) and ΔK values were obtained at K = 3. Utah and Montana were individualized in one cluster each, while Missouri, South Carolina, Ontario, Quebec and Bronx were predominantly assigned to the third cluster. Minnesota was intermediate, with approximately equal contributions of the three clusters (Fig. 3C). At K = 4 and K = 5, South Carolina and Bronx clustered separately.

Within Europe, lnP(D) increased with increasing K values and ΔK displayed two peaks, for K = 2 and K = 7. At K = 7, populations IT9, IT4, SE3, RO1 and UKR segregated in specific clusters, indicating their strong genetic divergence (Fig. 3C). Some other populations were predominantly assigned to the same clusters: B and D1 on the one hand, and F, IT1 and PO1 and the other hand. Finally, some populations in Central (SLO1, HU2, HU6, HU3) and Eastern Europe (PO2, UKR1, UKR2, RU4 and RU5) appeared highly admixed.

### Genetic Structure – F-Statistics

Multilocus fixation indices *F*_IS_ were significantly positive in all populations except Bronx and IT4 (Table 1). Nineteen populations displayed at least two significant monolocus tests, with up to four significant tests in Montana, Minnesota, Wuhan and Austral1. *F*_IS_ estimates were not significantly different between North America and Europe (P = 0.839), but were (marginally) significantly higher in non-European invasive populations than in North America and Europe (Table 1; P = 0.085 and P = 0.037, respectively).

At nuclear loci, the overall *F*_STn_ estimate was 0.073 (95% C.I.: 0.065–0.083). Analyses of Molecular Variance (AMOVAs) showed that the split between North America and Europe did not explain a significant proportion of the observed genetic variance either at nuclear or at chloroplast markers. Furthermore, though not significantly so, European populations showed somewhat greater among-population differentiation than did North American populations (*F*_STn_ = 0.065±0.006 and 0.054±0.012, respectively). At chloroplast loci, *F*_STcp_ = 0.411 and *N*_ST_ = 0.440 (not significantly different). In North America, *F*_STcp_ = 0.373 and was significantly lower than *N*_ST_ = 0.518 (P = 0.007). In contrast, in Europe, *F*_STcp_ = 0.389 and was not significantly different from *N*_ST_ = 0.384. These results showed a significant influence of the spatial component on the genetic structure in North America, but not in Europe.

**Figure 4:**
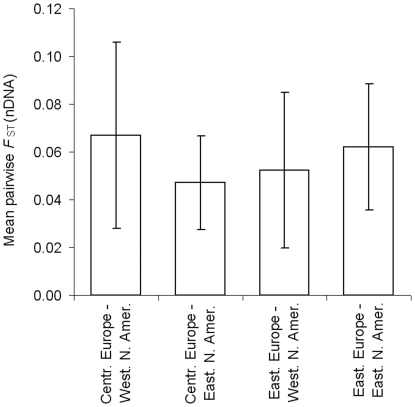
Figure 4. Mean pairwise *F*_ST_ indices (± S. D.) estimated at nDNA loci between populations from different regions. Regions include Central Europe (IT9 to PO1; 9 pops.), Eastern Europe (UKR to RU5; 5 pops.), western North America (Utah to Minnesota; 3 pops.) and eastern North America (Missouri to Bronx; 5 pops.).

Most exact tests of population differentiation were significant but some populations, in Central (SLO1, HU2, HU6, HU3) and Eastern Europe (PO2, UKR1, UKR2, RU4 and RU5), appeared clearly less differentiated from North American populations than the others (Table S2). We computed mean pairwise *F*_ST_ values among these two groups of low-differentiated European populations, to which we added clearly differentiated and geographically concomitant populations, and western and eastern North American populations, respectively. Standard deviations were large and differences were therefore not significant, but we observed the same pattern as in Structure: Central European populations were closer to eastern than to western North American populations while Eastern European populations were slightly closer to western North American than to eastern North American populations (Fig. 4). Populations B, F, D1 and PO2 were not included in these calculations because we observed incongruent results between Structure and *F*_ST_ estimates, and RO1 was also excluded because of its strong divergence.

**Figure 5:**
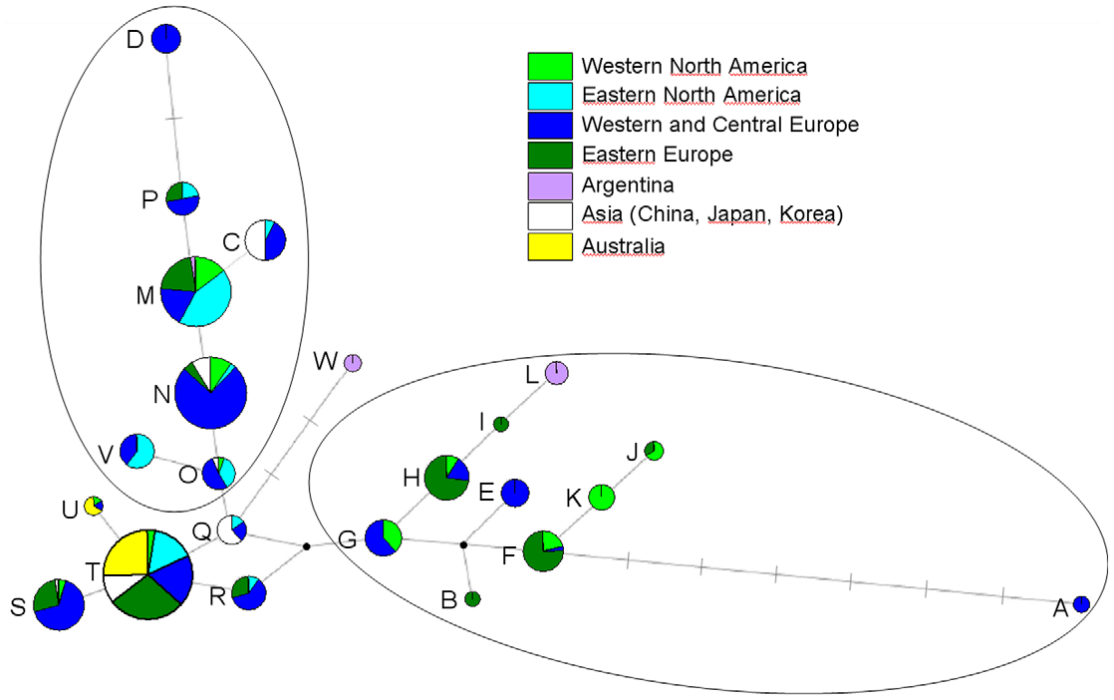
Figure 5. Median-joining network of cpDNA haplotypes. Ten haplotypes counting only one individual each (Table S1) were discarded, so that the network includes 23 haplotypes and 600 individuals. The size of each pie is proportional to the frequency of the corresponding haplotype. The colors indicate the geographical origin of the populations displaying each haplotype. Light green: western North America; light blue: eastern North America; dark green: Eastern Europe; dark blue: Western Europe. Purple: Argentina; White: Asia (China, Japan, Korea); Yellow: Australia. Black dots stand for unsampled haplotypes and each segment joining haplotypes represent one mutation. The two ellipses indicate the two areas of the network discussed in the text.

As for nDNA markers, there was no obvious geographical structure of the cpDNA genetic diversity at first sight. However, the close relationships of Utah with Eastern Europe, suggested by Bayesian clustering and *F*_ST_ indices based on nuclear DNA, was confirmed: Utah displays three cpDNA haplotypes, one of which was private and the other two that were otherwise mostly found in RU4 (haplotype H) and UKR (haplotype F; Fig. 5, Table S1). This was less clear for the Montana and Minnesota populations. Nevertheless, populations from western North America and Eastern Europe were predominantly represented in the right part of the network (which includes 53.3 and 54.6% of the samples of these two regions, respectively; Fig. 5), while populations from eastern North America and Western Europe were predominantly represented in the left part of the network (which includes 71.4 and 51.5% of the samples of these two regions [57.9% if excluding B, F, D1, PO2, RO1], respectively; Fig. 5).

**Figure 6:**
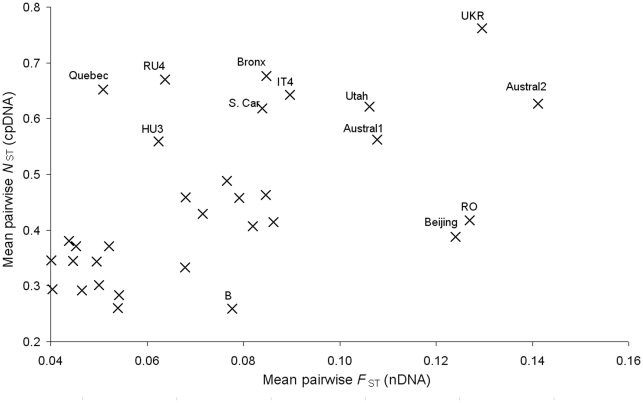
Figure 6. Relationship between mean pairwise differentiation indices at nDNA (*F*_ST_) and at cpDNA (*N*_ST_) loci. The correlation was significant (P = 0.002).

The nuclear- and chloroplast-based differentiation indices (*F*_STn_ and *N*_ST_, respectively) were significantly correlated, both when considering all pairs of populations (496 values, P<0.001) and when considering the mean pairwise differentiation indices for each population (32 values, P = 0.002; Fig. 6).

Within invasive populations, individuals were assigned to at least three (and up to seven) different source populations in North America (Table S3). The probabilities of assignment were less than 0.5 in 71% of the cases but, when only considering individuals with assignment probabilities above 0.5, samples from Western and Central Europe (populations IT9 to PO1, n = 47) were mostly assigned to populations from eastern North America (S. Car to Bronx; 68%) whereas individuals from Eastern Europe (UKR to RU4, n = 36) were mostly assigned to populations from western North-America (Utah, Montana and Minnesota; 58%).

**Figure 7:**
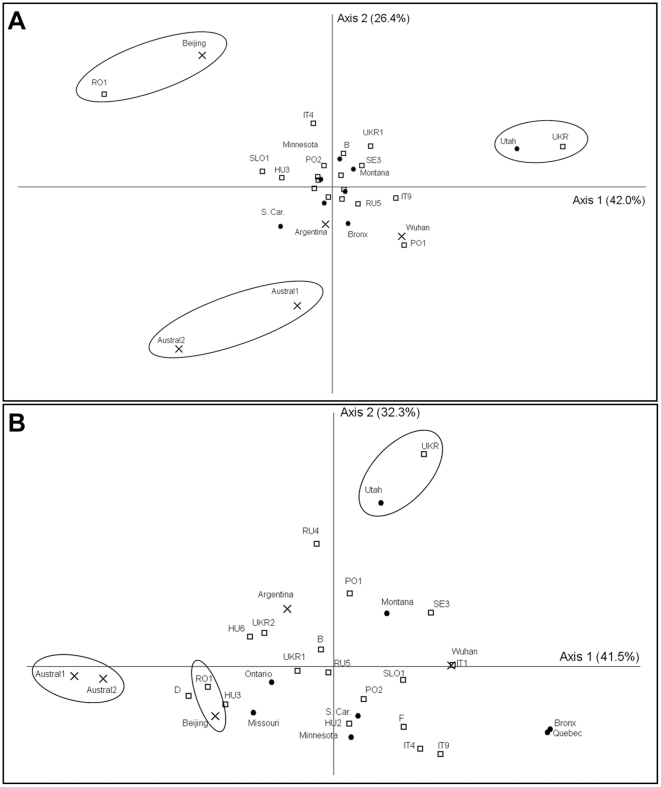
Figure 7. Principal Coordinate Analyses. A) At nDNA loci. B) At cpDNA loci. Dots: North America; open squares: Europe; crosses: non-European invasive populations. The percentages of variance explained by each axis are indicated within brackets.

Principal Coordinate Analyses allowed us to graphically represent the main patterns of genetic relationships, which were congruent with the results of the Bayesian clustering, haplotype network and differentiation indices: for both nuclear and chloroplast markers, the divergence of Austral1-Austral2 and Utah-UKR appeared clearly (Fig. 7A and 7B). Beijing and RO1 also appeared much differentiated for nuclear (but not for chloroplast) loci whereas Quebec and Bronx appeared more divergent at chloroplast than at nuclear loci (Fig. 7A and 7B).

Mantel tests revealed significant isolation by distance patterns in North America for both nuclear and chloroplast loci (P = 0.002 and P = 0.049, respectively; Fig. S1A) but not in Europe (P = 0.581 and P = 0.094, respectively; Fig. S1B).

## Discussion

Our results showed that most invasive populations were as diverse as the native populations. In Europe, Western and Central European populations were genetically more related to eastern North America, while Eastern European populations were closer to western North America. There was also a stark contrast between genetic structure in the native range, which displayed a clear geographic cline from East to West, and in Europe, where we detected no pattern of isolation by distance and only a weak influence of geography on the genetic structure.

### Our North American Sampling Does Not Encompass All Sources Of The Worldwide Invasion

The observation of some private alleles/haplotypes in invasive populations, the fact that some clusters inferred in Structure were virtually not represented in North America, and the low assignment probabilities of most invasive individuals to the North American sampled populations altogether suggested that our North American sampling does not encompass all sources of the worldwide (or even European) *A. artemisiifolia* invasion. An alternative explanation could be that some alleles/haplotypes were rare in the native area and that their frequency increased during introduction and subsequent invasion, but this scenario seems less parsimonious than the existence of unsampled source populations. A third possible explanation involves the *in situ* emergence of novel alleles/haplotypes following introduction, but this hypothesis appears even more unlikely: the worldwide expansion of common ragweed started in the mid- or late-XIXth century, i.e. at most 120–150 generations ago (since the plant is annual). The time scale of this study is thus much more restricted than in traditional phylogeographic studies, and the evolution of new alleles appears very improbable given the mutation rates at nuclear and chloroplast microsatellite loci (of the order of 10^−4^ and 10^−5^–10^−6^ mutation per locus per generation, respectively [36],[61]). This is even less probable when the private alleles/haplotypes diverge by more than one mutation from other alleles/haplotypes, since this would involve multiple mutation events.

### Multiple Introductions In Europe, Originating From (at Least) Two Distinct Regions In North America

We observed no significant loss of genetic diversity between North America and Europe, and European populations did not appear to have undergone recent bottlenecks (except population PO2). Furthermore, European populations were genetically differentiated (*F*_ST_ = 0.065 and most exact tests of differentiation were significant). Because introductions almost always involve sampling and founder events, we consider it improbable that populations in the introduced range could have arisen from single population introductions and still retain this high amount of genetic diversity. This leaves us with two alternatives: i) populations were founded by multiple colonisations from different populations in the native range, as suggested by the assignment test and previous findings of extremely high allelic diversity in the introduced range [30] or ii) introduced populations arose from independent introductions from single source populations and subsequent gene flow has restored diversity to similar levels as that found in the native range. We note, however, that the genetic differentiation of European populations suggests low gene flow. Furthermore, for restoring genetic diversity, gene flow must have involved (human-mediated) long distance dispersal and not natural processes of pollen and seed exchange between neighbouring populations, since this would have left a trace of isolation by distance, for which we found no evidence among European populations. Therefore, we favour the scenario involving multiple introductions at both the population and the regional scales. This study thus adds to the pre-existing body of evidence that multiple introductions seem to be a common feature of biological invasions [9],[14],[62].

Overall, Western and Central European populations seemed more related to eastern North American populations whereas Eastern European (Ukrainian and Russian) populations were genetically more similar to western North American populations. This clarifies the pattern observed by Gladieux et al. [35], who found that eastern North American and French populations were clearly differentiated from Eastern European populations. However, they could not interpret this result further since they did not include populations from Western North America. The same kind of geographic pattern was observed in the invasive grass *Bromus tectorum* [13]: the authors detected some genetic similarity between eastern Canada and Germany-Czech Republic on the one hand, and between western Canada and Hungary-Slovakia on the other. These results together strengthen the idea that differences in commercial exchange between different regions of North America and Europe have influenced sources of invasive populations.

We also observed that the two groups of populations in Slovenia-Hungary and Ukraine-Russia were less differentiated, more diverse and more admixed than most other European populations. This may indicate more frequent colonization events than in other regions, and/or higher ongoing gene flow among populations. Interestingly, these populations are located in some of the most heavily infested countries in Europe, i.e. Hungary and Russia. The French population was also sampled in a region where common ragweed is a very aggressive invader, and was also found to have low differentiation and high diversity. Whether higher genetic diversity increases invasion success or whether areas where an invasion is particularly successful (containing high number of populations) leads to high genetic diversity remains uncertain, but would deserve further investigations: genetic diversity has long been considered a prerequisite for invasion success because of the assumed correlation between variation at neutral markers and adaptive potential (e.g. [11],[12]), but this now appears controversial (see e.g. [63]).

### Fewer Introduction Events In Non-European Invasive Populations

In contrast to Europe, other invasive populations displayed reduced genetic diversity and a trend towards increased within-population *F*_IS_ indices compared to the native area. This suggests that introduction events may have been less frequent and involved a lower number of differentiated source populations and/or individuals (i.e. lower propagule pressure), possibly leading to lower population sizes and more genetic drift (but only the Beijing population seems to have undergone a recent bottleneck). In turn, this could cause increased rates of selfing and/or inbreeding (i.e. crosses between related plants), explaining the slightly higher *F*_IS_ estimates (see below for a discussion on *F*_IS_ estimates). Such a pattern would be consistent with greater commercial isolation from North America and less military exchange with North America than was the case for Europe during the two World Wars, and/or better quarantine procedure (e.g. in Australia, where the species is quite restricted).

The two Australian populations were genetically similar and strongly differentiated from all other populations. They most likely originated from an unsampled source, either through a single introduction event followed by dispersal within Australia (probably from Austral1 to Austral2 since Austral1 exhibits a slightly higher genetic diversity), or through two independent introduction events from the same source population(s). A similar pattern was observed for the Beijing and Romanian populations, which were closely related but highly differentiated from all others. The genetic similarity between Romania and Beijing populations may be explained either by commercial trade with the same (unsampled) region(s) in North America, or by a secondary introduction from Romania to China (since we observed lower diversity in Beijing).

The other non-European invasive populations, Wuhan and Argentina, were less differentiated from North America and Europe. These populations were also slightly more diverse than Australian and Beijing populations, indicating that they probably experienced more introduction events or less strong founder effects.

### Genetic Differentiation Is Influenced By Geography In North America, But Not In Europe

We observed similar levels of population differentiation in North America and Europe (*F*_STn_ = 0.054 vs. 0.065, respectively) and the slightly stronger genetic structure in Europe may result from i) the fact that potentially divergent North American populations were missing from our sampling and ii) the establishment, by chance, of different genotypes in different areas following multiple introductions (as shown in *Centaurea diffusa* [64]).

Most importantly, we observed a major difference between North America and Europe in how genetic diversity was structured geographically, which gave an insight into the colonization process in Europe: i) we found significant isolation by distance (i.e. a positive correlation between genetic and geographic distances) in North America but not in Europe; ii) although we could identify two groups of European populations originating from distinct source regions, the pattern was not clear-cut and did not include all sampled populations, and the overall genetic structure was much more geographically organised in North America than in Europe (based on the Structure results); and iii) distinct haplotypes were significantly more related within populations than among populations in North America (*N*_STcp_ > *F*_STcp_) but not in Europe. All these results were in agreement and, first, show that North American populations are at migration-drift equilibrium whereas European populations are not. Second, they indicate that range expansion in Europe occurred by a series of long-distance dispersal events and the establishment of outlying populations, similarly to what was found in e.g. invasive *Heracleum* taxa in Europe [65] and *Centaurea diffusa* in North America [64], instead of a simple advancing wave front with stepwise colonisation events. Long distance dispersal events were probably human-mediated, and may have involved both transatlantic and within-Europe dispersal.

Whereas the installation of new populations obviously required seed dispersal and establishment, most subsequent gene dispersal seems mediated through pollen, as indicated by the much stronger among-population differentiation at cpDNA (only dispersed by seeds) than at nDNA (dispersed by both seeds and pollen) markers. This is congruent with previous knowledge on pollen dispersal in *A. artemisiifolia*, which can reach hundreds of kilometres (although the duration of pollen viability is unknown [19],[66]).

### Genetic Structure At The Worldwide Scale

Genetic differentiation at the worldwide scale was low. This may be explained by weak founder effects when the species was introduced, on-going gene flow, and/or insufficient time for genetic drift to differentiate the populations since their establishment. In addition, there was little spatial component to the genetic structure: the geographic split between North America and Europe explained no significant part of the total genetic variance. Also, the combined use of several Bayesian algorithms allowed the delineation of five genetically-based clusters, but these clusters could not be related to clear geographic entities.

### Within-Population Genetic Structure And Mating System

Almost all within-population fixation indices *F*_IS_ were significant, suggesting a deficit in heterozygotes. This was confirmed by the software Instruct, which estimated inbreeding coefficients of 0.3–0.4 within clusters. Although this result was congruent with previous population genetic studies of *A. artemisiifolia* [29],[30],[35], it was surprising because the species has been shown to be outcrossing and self-incompatible [19]. We hypothesise that selfing and/or biparental inbreeding, as well as a spatial substructuring within populations (i.e. Wahlund effect) may be involved.

In earlier genetic surveys [29],[30],[35], null alleles were proposed as the most likely explanation for positive *F*_IS_ estimates. Although we do not exclude this possibility, we do not favor it for several reasons. First, we did not observe any repeated amplification failure for any given locus in any population (which is expected with null alleles since homozygotes for a null allele will produce no PCR amplification). Therefore, even if there are some null alleles, they occur at very low frequencies and contribute very little to overall heterozygosity deficit (and therefore to *F*_IS_ calculations). Second, in our study, most populations displayed significant deficit of heterozygotes at several loci and significant monolocus *F*_IS_ estimates were widely distributed across loci. Third, Genton et al. [30] and Gladieux et al. [35] used the same five nuclear microsatellite markers, which totally differ from the nine markers used by Chun et al. [29], but both groups of loci lead to positive *F*_IS_ values (in the present study, we used three markers in common with Genton et al. [30] and Gladieux et al. [35], and five markers in common with Chun et al. [29]). This would mean that a high number of loci display null alleles, which does not seem very plausible. Fourth, Gladieux et al. [35] explained high *F*_IS_ in Eastern Europe by the fact that microsatellites were developed on French populations and that the genetic divergence of Eastern Europe may explain the occurrence of some mutations at primer sites, leading to null alleles. However, they also documented very high *F*_IS_ estimates in France (mean *F*_IS_ of 0.490 ± 0.0469). Altogether, these lines of evidence suggest that null alleles are probably not the main cause for the observed deficits of heterozygotes within populations.

All *F*_IS_ estimates were quite similar and there was no evidence for an evolutionary shift towards higher selfing rates in the introduced range, as has been suggested as a general pattern in invasive species [67]. A Wahlund effect is possible in the sampled populations given the very large size of most of them (sometimes counting more than 10000 plants and covering areas of several thousand square meters). Nevertheless, further studies on the breeding system of *A. artemisiifolia* and its potential variation across populations (or regions) would be interesting to better understand these positive *F*_IS_ estimates. Such studies appear especially needed since *A. artemisiifolia* was long reported as self-compatible [15],[68] and Friedmann & Barrett [19], who showed the self-incompatibility in Canadian populations, acknowledged the possibility that some other populations may exhibit partial self-compatibility. Moreover, based on controlled pollinations, observations of pollen-tube growth and allozyme analyses in three populations from China, Li et al. [69] concluded that selfing was possible (although leading to lower seed sets than outcrossing) and estimated an average selfing rate of 0.22.

### Conclusions

The present study shows how variable the history of distinct (but sometimes geographically close) invasive populations can be. This highlights the importance of sampling as many populations as possible to avoid biased inferences (see also [70]). It also appears desirable to sample with no major geographic gap, especially in the native range. Gladieux et al. [35], although with more populations from the native area than in the present survey, had poorer geographic coverage and could only conclude that Eastern European and French populations did not originate from the same source populations. Our geographically larger sampling area allowed us to document this pattern more precisely and propose possible source regions of the Eastern European *A. artemisiifolia* populations.

We showed that *A. artemisiifolia* was introduced multiple times in most parts of its invasive range, leading to high levels of within-population and regional diversity. In Europe, introduction events probably mainly involved two different regions of the native area, with populations of Central Europe originating from eastern North America, and populations of Eastern Europe originating from more western North America. Our results indicate that the expansion of the European range mostly occurred through long-distance seed dispersal, explaining the weak association between genetic differentiation and geographic location in this area (in contrast to the native range, where isolation by distance was observed). Finally, heterozygote deficiencies may be explained by a Wahlund effect, but further investigations on the breeding system would provide useful information to better explain this result.

Such data offer opportunities to study the ecological and/or evolutionary changes involved in the invasion process (e.g. [12],[71]), and may help to predict the potential further expansion of the species. *A. artemisiifolia* exhibits latitudinal variation in flowering phenology both in the native range [72] and in invasive populations in China, which may indicate on-going local adaptation and allow further expansion northwards of the invasive populations [69]. The same may be true in Europe, where the species is increasingly often observed flowering in Scandinavia, in spite of the short growing season [73]. The mechanisms underlying such potential, rapid adaptive processes, and their consequences would be worth examining more in depth, and in relation with global warming. Genetic data can also benefit the development of effective prevention and management strategies. More globally, this study adds to the growing body of data on the genetic patterns and processes involved in biological invasions, which will hopefully lead to an increased understanding and better management in order to minimize their negative impacts on biodiversity, economy, and also human health in the case of *A. artemisiifolia*.

## Supporting Information

Figure S1 Relationship between geographic and genetic distances between all pairs of populations based on nDNA loci. **A**) In North America (P  =  0.002). B) In Europe (P =  0.581). The geographic distance was expressed as the log_10_ of interpopulation distance in km; the genetic distance was expressed as *F*_ST_/(1-*F*_ST_).
(TIF)

Table S1 CpDNA haplotypic composition of the studied populations. Populations are grouped into three spatial groups (North America, Europe and non-European invasive populations) and roughly ordered from West to East. Haplotypes are ordered from the most frequent to the least frequent. Haplotypes that are in bold are private to one population.
(XLS)

Table S2 Pairwise *F*_ST_ indices among all pairs of populations, estimated at nDNA loci. Populations are grouped into three spatial groups (North America, Europe and non-European invasive populations) and roughly ordered from West to East. *F*_ST_ estimates that are highlighted in grey correspond to non-significant exact tests of differentiation.
(XLS)

Table S3 Results of the assignment test of invasive populations to North American populations, based on nDNA loci. The number of individuals assigned to each North American population is given, either considering all individuals or only individuals with assignment probabilities above 0.5.
(XLS)
