# Supplementary figures and images for: Nuclear and Chloroplast Microsatellites Show Multiple Introductions in the Worldwide Invasion History of Common Ragweed, Ambrosia artemisiifolia
Source: PLoS One. 2011 Mar 10;6(3):e17658. doi: 10.1371/journal.pone.0017658 (PMC3053376; doi:10.1371/journal.pone.0017658)

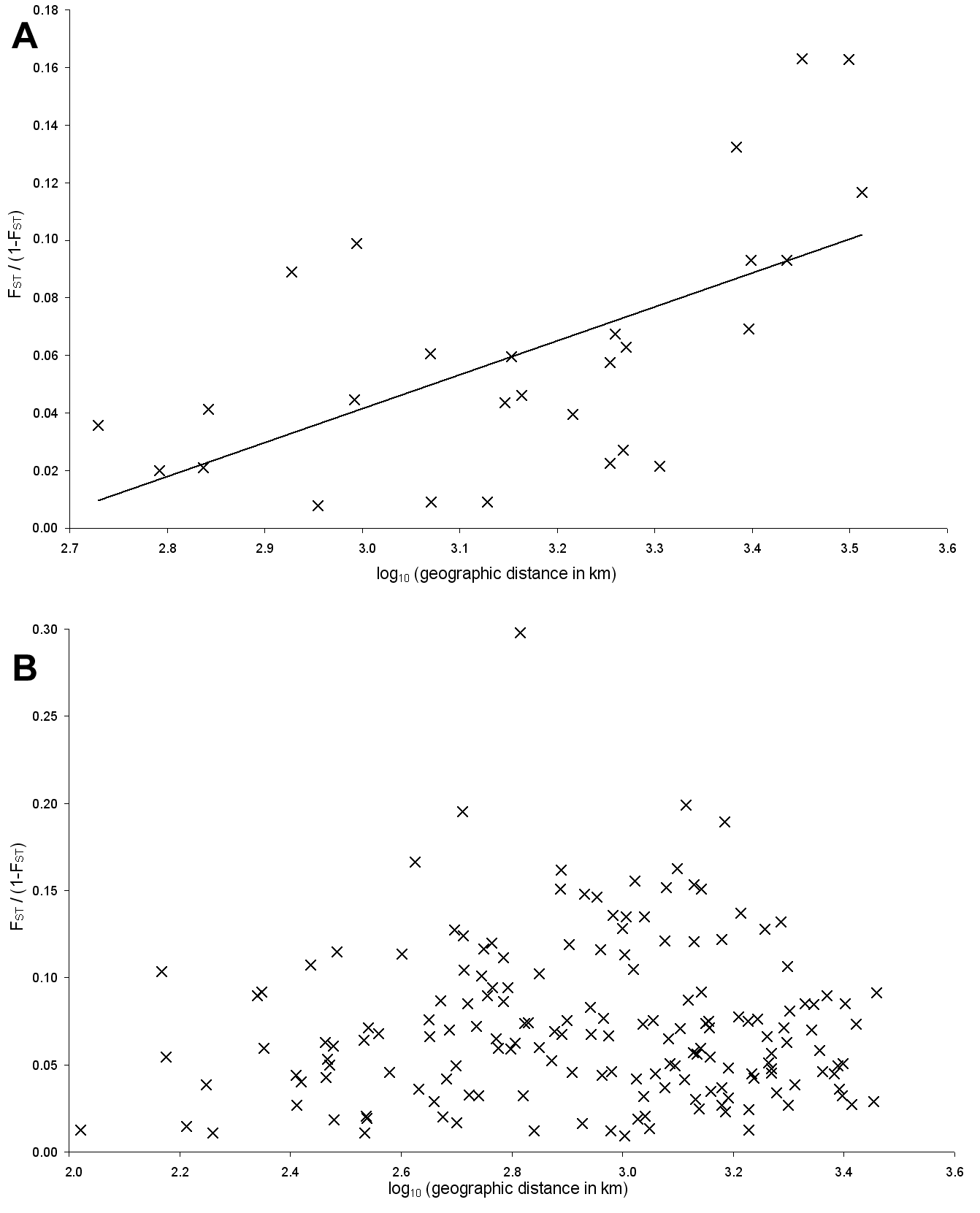

Supplement: Figure S1 — In North America (P = 0.002). B) In Europe (P = 0.581). The geographic distance was expressed as the log10 of interpopulation distance in km; the genetic distance was expressed as F ST/(1-F ST). (TIF) [file pone.0017658.s001.tif]
